# Rapamycin Perfluorocarbon Nanoparticle Mitigates Cisplatin-Induced Acute Kidney Injury

**DOI:** 10.3390/ijms24076086

**Published:** 2023-03-23

**Authors:** Qingyu Zhou, James D. Quirk, Ying Hu, Huimin Yan, Joseph P. Gaut, Christine T. N. Pham, Samuel A. Wickline, Hua Pan

**Affiliations:** 1 Taneja College of Pharmacy, University of South Florida, Tampa, FL 33620, USA; 2 Mallinckrodt Institute of Radiology, Washington University School of Medicine, St. Louis, MO 63110, USA; 3 Division of Rheumatology, Washington University School of Medicine, St. Louis, MO 63110, USA; 4 Department of Medicine, Washington University School of Medicine, St. Louis, MO 63110, USA; 5 Department of Pathology and Immunology, Washington University School of Medicine, St. Louis, MO 63110, USA; 6 Morsani College of Medicine, University of South Florida, Tampa, FL 33620, USA; 7 Department of Biomedical Engineering, Washington University in St. Louis, St. Louis, MO 63130, USA

**Keywords:** cisplatin, acute kidney injury, rapamycin, perfluorocarbon nanoparticles, fluorine magnetic resonance imaging, magnetic resonance spectroscopy, autophagy, inflammation, apoptosis

## Abstract

For nearly five decades, cisplatin has played an important role as a standard chemotherapeutic agent and been prescribed to 10–20% of all cancer patients. Although nephrotoxicity associated with platinum-based agents is well recognized, treatment of cisplatin-induced acute kidney injury is mainly supportive and no specific mechanism-based prophylactic approach is available to date. Here, we postulated that systemically delivered rapamycin perfluorocarbon nanoparticles (PFC NP) could reach the injured kidneys at sufficient and sustained concentrations to mitigate cisplatin-induced acute kidney injury and preserve renal function. Using fluorescence microscopic imaging and fluorine magnetic resonance imaging/spectroscopy, we illustrated that rapamycin-loaded PFC NP permeated and were retained in injured kidneys. Histologic evaluation and blood urea nitrogen (BUN) confirmed that renal structure and function were preserved 48 h after cisplatin injury. Similarly, weight loss was slowed down. Using western blotting and immunofluorescence staining, mechanistic studies revealed that rapamycin PFC NP significantly enhanced autophagy in the kidney, reduced the expression of intercellular adhesion molecule 1 (ICAM-1) and vascular cell adhesion molecule 1 (VCAM-1), as well as decreased the expression of the apoptotic protein Bax, all of which contributed to the suppression of apoptosis that was confirmed with TUNEL staining. In summary, the delivery of an approved agent such as rapamycin in a PFC NP format enhances local delivery and offers a novel mechanism-based prophylactic therapy for cisplatin-induced acute kidney injury.

## 1. Introduction

Cisplatin remains one of the most broadly used anticancer treatments and is prescribed as first-line chemotherapy for about 10–20% of cancer patients [1]. In current medical practice, patients taking cisplatin typically receive multiple serial doses. It has been reported that a 5-day consecutive cisplatin dosing regimen at 15–20 mg/m^2^/day results in acute kidney injury in 50–70% of patients [2]. This dose-dependent organ toxicity, particularly nephrotoxicity [3,4,5], thus limits the cumulative administered dose and often forces patients to stop this effective therapy. Even ten years after recovered from cisplatin-induced acute kidney injury, patients still face a higher risk of developing chronic kidney diseases and increased risk of mortality [6,7,8]. Clearly, the adverse effects from cisplatin treatment can be prevalent, severe, and long-lasting. Because cisplatin-induced nephrotoxicity is managed only by supportive measures in current medical practices, it is critical to develop specific strategies to address this commonly encountered medical problem.

The benefits of enhancing autophagy in early-stage cisplatin-induced acute kidney injury are well recognized [9,10,11,12,13,14]. Transgenic mice with specific knockout of either Atg5 [15] or Atg7 [16] in the proximal tubule are more susceptible to cisplatin-induced acute kidney injury compared to wild-type mice. However, in the repair stage, enhanced autophagy may impair cell proliferation and impede recovery [17]. Although effective interventions for cisplatin-induced acute kidney injury remain to be developed based on mechanistic rationales [18,19], compelling experimental evidence suggests that enhanced autophagy [9,12,13] in concert with anti-inflammatory strategies [18] could result in renoprotective effects during early-stage cisplatin-induced injury.

Rapamycin is a potent autophagy inducer signaling through mTOR, which initiates a cell survival program to recycle amino acids by depredating long-lived proteins and dysfunctional organelles [20]. Rapamycin can also suppress downstream inflammation through inhibition of mTORC1 [21,22,23,24,25]. In a previous study of mild kidney injury induced by injecting cisplatin at 10 mg/kg, we observed that rapamycin PFC NP outperformed free rapamycin in protecting renal function at 48 h [26]. Here, we sought to define the potential utility of rapamycin PFC NP in mitigating more severe acute kidney injury induced by cisplatin treatment at 25 mg/kg. Our results demonstrated that rapamycin PFC NP is effective for mitigating kidney injury under these circumstances. At the molecular level, renal protection was accompanied by autophagy enhancement, vascular inflammation reduction, and apoptosis suppression.

## 2. Results

### 2.1. Accumulation of Rapamycin PFC NP in Kidney after Systemic Administration

**Figure 1:**
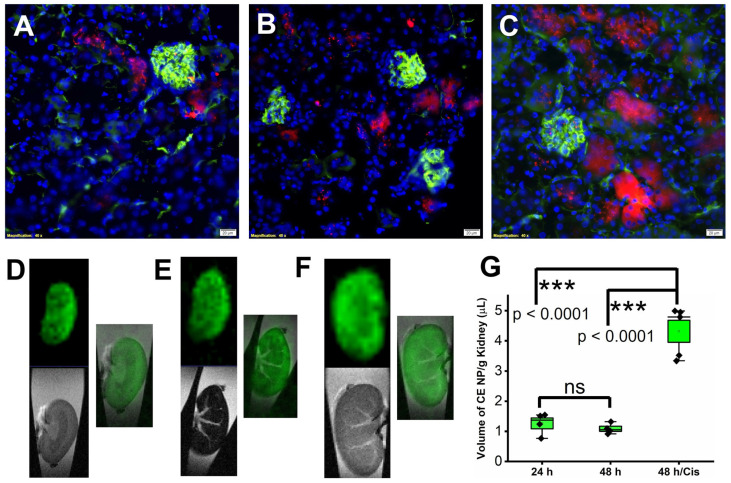
Delivery of PFC NP to the kidney. Representative fluorescent microscopy images illustrate delivery of PFC NP (red) to kidneys from groups 1 to 3, with blood vessels defined by lectin staining (green) (**A**–**C**). DAPI nuclear staining is shown in blue. The size of the scale bar is 20 µm and the magnification is 40×. (**D**–**F**) Representative ^19^F MRI (pseudo color—green) and ^1^H MRI (grey scale) as well as their overlay of the kidneys from groups 1–3. (**G**) ^19^F MRS quantification of nanoparticles delivered to the kidneys. (*n* = 4, 24 h; *n* = 4, 48 h; and *n* = 5 48 h/Cis). ns: not significantly different. Results are presented as mean ± SEM.

To evaluate the delivery of PFC nanoparticles to the kidney, three groups of mice were utilized. All mice received 1 mL/kg retroorbital injection of crown ether (CE) core PFC NP with the fluorophore, Rhodamine, conjugated on PFC NP’s lipid monolayer. After NP injection, mice were treated as follows: Group 1 (“24 h”) were euthanized 24 h later; Group 2 (“48 h”) were euthanized 48 h later; and Group 3 (“48 h/Cis”) received 25 mg/kg cisplatin treatment 24 h after NP injection and were euthanized 48 h after NP injection. Ten minutes before euthanasia, all mice received Lycopersicon Esculentum lectin with DyLight 488 (green) to delineate the renal vasculature. Following euthanasia, mice were systemically perfused with saline before the kidneys were collected for fluorine (^19^F) MRI/MRS and fluorescent microscopic evaluations. As illustrated in Figure 1A–C (groups 1–3), the green fluorescent signal highlighted the glomeruli and blood vessels in the kidney. The red fluorescent signal originating from Rhodamine illustrated the location of PFC NP in the kidney.

To validate that the red fluorescent signal emanated from intact PFC NP instead of shed Rhodamine fluorophore, ex vivo ^1^H and ^19^F MRI were performed on excised kidneys. As illustrated in Figure 1D–F (obtained from kidneys in group 1–3, respectively), ^1^H MRI (grey scale image) delineated the location of the kidneys in the test tubes and ^19^F MRI (pseudo color green image of the ^19^F from the fluorine core of the PFC NP) registered the position of the nanoparticles. Consistent with the microscopic fluorescent images of the kidneys (Figure 1A–C), the overlayed ^1^H and ^19^F MRI confirmed the delivery of intact CE nanoparticles to the kidneys from all three groups. For quantifying the delivered CE nanoparticles, ^19^F MRS was performed. As shown in Figure 1G, the amounts of CE nanoparticles detected were 1.26 ± 0.18, 1.09 ± 0.09, and 4.32 ± 0.37 µL/g of kidney weight from groups 1 to 3, respectively. The results demonstrated that cisplatin treatment increased the accumulation of CE NP about 3-fold compared to no treatment.

### 2.2. Rapamycin PFC NP Treatment Mitigated Renal Dysfunction Induced by Cisplatin

**Figure 2:**
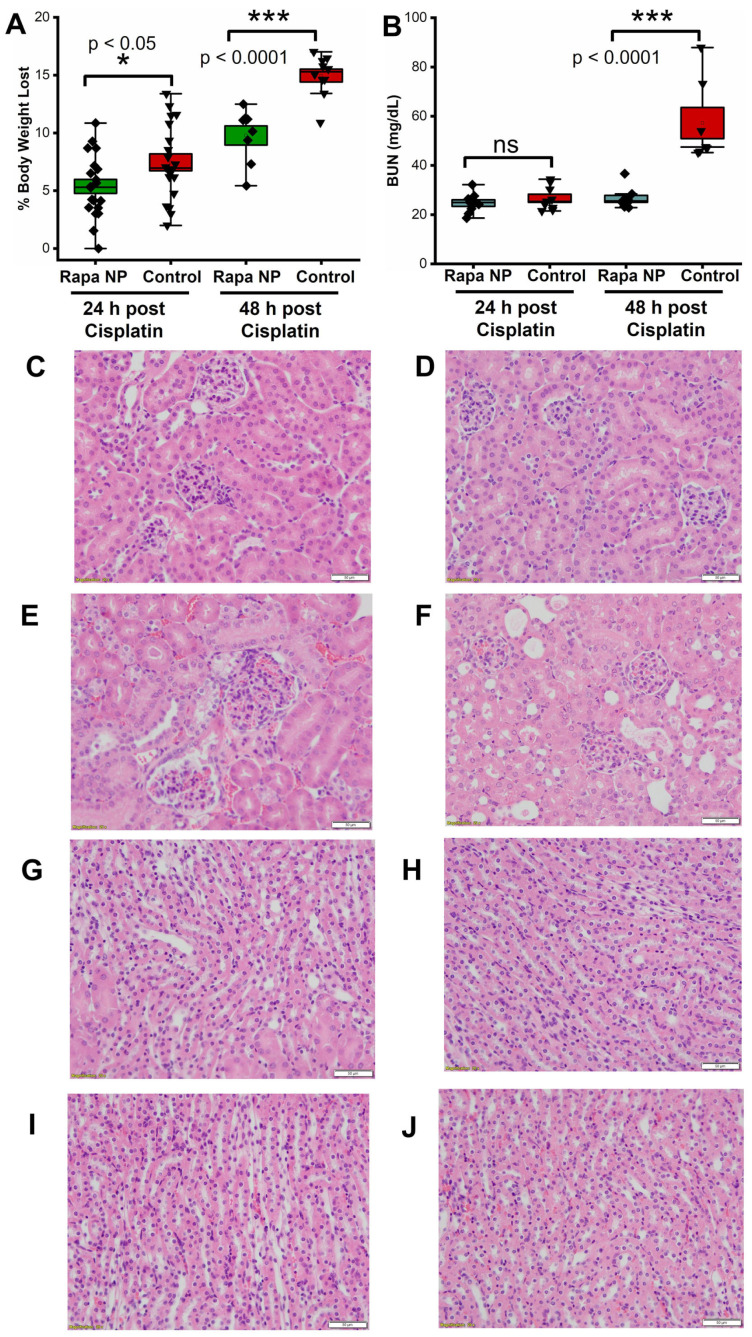
Rapamycin PFC NP treatment mitigated cisplatin-induced kidney damage. (**A**) Rapamycin PFC NP treatment prevented cisplatin-induced body weight loss (*n* = 17 Rapa NP 24 h post Cisplatin; *n* = 17 Control 24 h post Cisplatin; *n* = 8 Rapa NP 48 h post Cisplatin; and *n* = 10 Control 48 h post Cisplatin). (**B**) Rapamycin preserved renal function from cisplatin-induced acute kidney injury (*n* = 9 Rapa NP 24 h post Cisplatin; *n* = 9 Control 24 h post Cisplatin; *n* = 9 Rapa NP 48 h post Cisplatin; and *n* = 7 Control 48 h post Cisplatin). Representative H&E images of kidney cortex from mice treated with rapamycin NP and euthanized at 24 h (**C**) or 48 h (**D**) after cisplatin injection, as well as control mice euthanized 24 h (**E**) or 48 h (**F**) after cisplatin injection. Representative H&E images of kidney medullas from mice treated with rapamycin NP and euthanized at 24 h (**G**) or 48 h (**H**) after cisplatin injection, as well as control mice euthanized 24 h (**I**) or 48 h (**J**) after cisplatin injection. The size of the scale bar is 50 µm and the magnification is 20×. ns: not significantly different. Results are presented as mean ± SEM.

To determine if rapamycin PFC NP ameliorates cisplatin-induced acute kidney injury, 34 mice received either rapamycin NP treatment or saline (control) 24 h prior to i.p. cisplatin injection at a dose of 25 mg/kg and were euthanized either 24 or 48 h after cisplatin injection. The body weights of the mice were monitored daily throughout the experimental period. As demonstrated in Figure 2A, rapamycin NP treatment significantly reduced body weight loss after cisplatin injection both at 24 h (5.38 ± 0.68% vs. 8.08 ± 0.73%, rapamycin NP vs. control, *p* < 0.05) and 48 h (9.79 ± 0.83% vs. 14.97 ± 0.56%, rapamycin NP vs. control, *p* < 0.0001).

For evaluation of kidney function, blood urea nitrogen (BUN) measurements were performed. The BUN levels at 24 h after cisplatin injection were 24.71 ± 1.34 mg/dL vs. 26.67 ± 1.62 mg/dL for rapamycin PFC NP-treated vs. untreated controls (*p* > 0.05). The BUN values of the rapamycin PFC NP-treated mice were significantly lower than those of the control mice at 48 h after cisplatin injection (26.43 ± 1.41 mg/dL vs. 57.23 ± 6.33 mg/dL; *p* < 0.0001, Figure 2B). The normal range for murine BUN is 17–28 mg/dL [27], which confirmed that rapamycin PFC NP treatment mitigated renal damage.

Microanatomical consequences of cisplatin injury were depicted in the H&E images, which were evaluated by a renal pathologist who was blinded to the treatment of all four groups. No visible injury was observed in the kidney cortex region obtained 24 h after cisplatin injection with or without rapamycin PFC NP treatment (Figure 2C,E) or in the kidneys from mice treated with rapamycin PFC NP and euthanized 48 h after cisplatin injection (Figure 2D). However, the cortex in the kidneys from untreated control mice 48 h after cisplatin injection exhibited focal tubular injury, focal tubular epithelial cell cytoplasmic vacuolization, and cell sloughing (Figure 2F). Different from the renal cortex region, where proximal tubule cells are located, the renal medulla region did not exhibit remarkable injuries in the cross-treated and control groups over the two evaluated time points (Figure 2G–J).

### 2.3. Rapamycin PFC NP Enhanced Autophagy in Kidney

**Figure 3:**
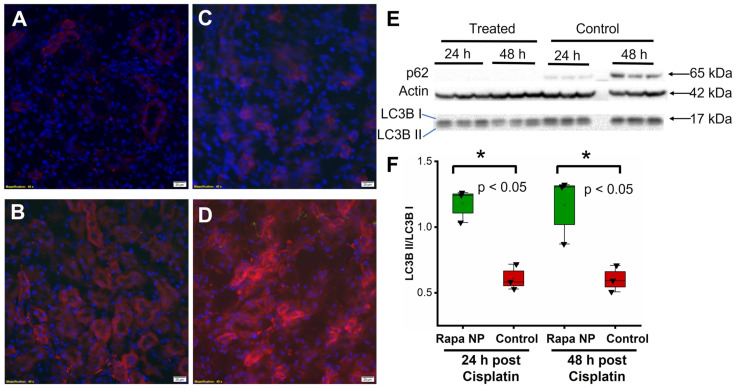
Rapamycin PFC NP enhanced autophagy in the kidney. Representative images of p62 staining (red) in the kidney from mice 24 h after cisplatin injection with (**A**) or without (**B**) rapamycin PFC NP treatment. Representative images of p62 staining (red) in the kidney from mice 48 h after cisplatin injection with (**C**) or without (**D**) rapamycin PFC NP treatment. DAPI nuclear staining is shown in blue. The size of the scale bar is 20 µm and the magnification is 40×. (**E**) Western blots of p62, LC3B I, and LC3B II indicated that rapamycin PFC NP treatment reduced p62 levels and increased the LC3B II/I ratio. β-Actin served as the loading control. (**F**) Quantification of LC3B II/I. (*n* = 3 per group, mean ± SEM).

To determine the autophagy response to rapamycin PFC NP, p62 staining was performed on kidneys of mice obtained either 24 or 48 h after cisplatin injection with or without rapamycin PFC NP treatment. An enhancement in autophagy would be expected to deplete or reduce p62 levels as this protein links autophagy and ubiquitin-proteosome pathways and is used in the process of protein recycling. The results showed that p62 levels in the kidneys of rapamycin PFC NP-treated mice either 24 h (Figure 3A) or 48 h (Figure 3C) after cisplatin injection were significantly lower than those in untreated control mice either 24 h (Figure 3B) or 48 h (Figure 3D) after cisplatin injection. The observation was confirmed by Western blotting (Figure 3E), where p62 was not detectable in the kidneys of rapamycin PFC NP-treated mice whereas p62 levels in the kidneys of untreated control mice 48 h after cisplatin injection were much higher. These results suggested that rapamycin PFC NP enhanced autophagy.

Western blotting of the LC3B II/I ratio further confirmed the enhancement of autophagy following rapamycin PFC NP treatment. An enhancement in autophagy would be expected to increase the LC3B II/I ratio because LCB3 I is converted to LC3B II in the process. As shown in Figure 3F, the LC2B II/I ratio in the kidneys of mice treated with rapamycin PFC NP 24 h after cisplatin injection was 1.18 ± 0.07 compared to 0.61 ± 0.05 for untreated controls (*p* < 0.05), which was a 93% increase. Forty-eight hours after cisplatin injection, the LC3B II/I ratios were 1.17 ± 0.15 for rapamycin PFC NP-treated mice and 0.60 ± 0.06 for untreated controls (*p* < 0.05), which was a 95% increase. Therefore, rapamycin PFC NP enhanced kidney autophagy.

### 2.4. Rapamycin PFC NP Treatment Suppressed Renal Endothelial Inflammation

**Figure 4:**
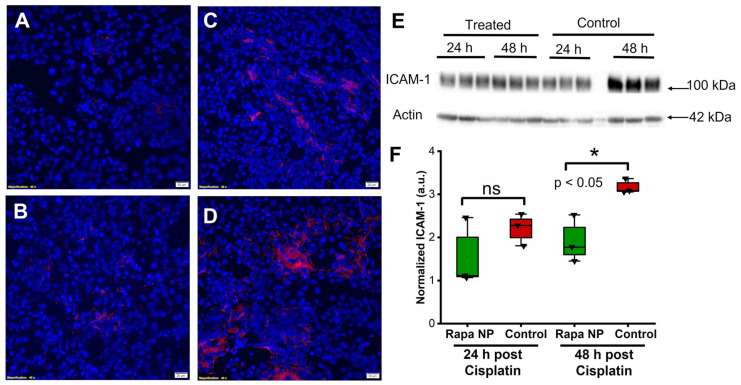
Rapamycin PFC NP treatment reduced ICAM-1 expression in the kidney. (**A**,**B**) Representative images of ICAM-1 staining (red) in the kidney from mice 24 h after cisplatin injection with (**A**) or without (**B**) rapamycin PFC NP treatment. (**C**,**D**) Representative images of ICAM-1 staining (red) in the kidney from mice 48 h after cisplatin injection with (**C**) or without (**D**) rapamycin PFC NP treatment. DAPI nuclear staining is shown in blue. The size of the scale bar is 20 µm and the magnification is 40×. (**E**) Western blot of ICAM-1 showing that rapamycin PFC NP treatment reduced ICAM-1 expression 48 h after cisplatin injection. β-Actin served as the loading control. (**F**) Quantification of ICAM-1 expression. ns: not significantly different. (*n* = 3 per group, mean ± SEM).

The endothelial cellular adhesion molecules, intercellular adhesion molecule 1 (ICAM-1) and vascular cell adhesion molecule 1 (VCAM-1), play a crucial role in mediating leukocyte adhesion to vascular endothelial cells and tissue inflammation. Accordingly, the effects of rapamycin PFC NP on the expression of ICAM-1 and VCAM-1 were investigated by immunofluorescent staining and western blotting. ICAM-1 staining was performed on kidneys from mice either 24 or 48 h after cisplatin injection with or without rapamycin PFC NP treatment. The results illustrated that the ICAM-1 signals from the kidneys of rapamycin PFC NP-treated mice (Figure 4A) was lower than control mice (Figure 4B) at 24 h after cisplatin injection. While the ICAM-1 expression was significantly reduced in treated mice h (Figure 4C) comparing to control mice (Figure 4D) 48 after cisplatin injection. The reduction in ICAM-1 expression was confirmed by western blotting (Figure 4E). As demonstrated in Figure 4F, in the kidneys from mice 24 h after cisplatin injection, ICAM-1 protein expression normalized to β-actin was 1.55 ± 0.46 for rapamycin PFC NP-treated mice vs. 2.21 ± 0.22 for untreated control mice (*p* > 0.05). Forty eight hours after cisplatin injection, ICAM-1 protein expression normalized to β-actin was 1.92 ± 0.32 for rapamycin PFC NP-treated mice vs. 3.17 ± 0.10 for untreated control mice (*p* < 0.05), which represented a 39% decrease.

**Figure 5:**
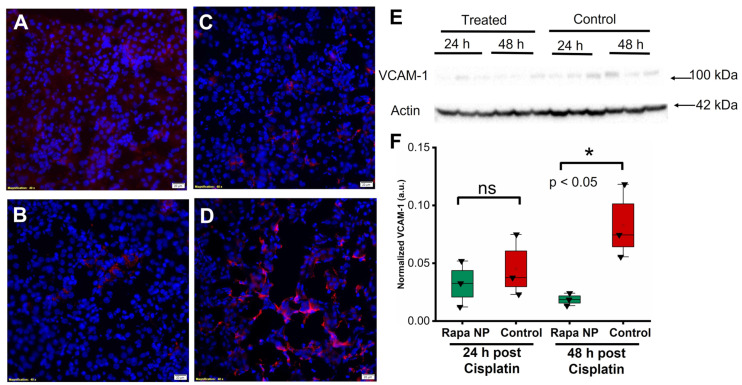
Rapamycin PFC NP treatment reduced VCAM-1 expression in the kidney. (**A**,**B**) Representative images of VCAM-1 staining (red) in the kidney from mice 24 h after cisplatin injection with (**A**) or without (**B**) rapamycin PFC NP treatment. (**C**,**D**) Representative images of VCAM-1 staining (red) in the kidney from mice 48 h after cisplatin injection with (**C**) or without (**D**) rapamycin PFC NP treatment. DAPI nuclear staining is shown in blue. The size of the scale bar is 20 µm and the magnification is 40×. (**E**) Western blot of VCAM-1 showing rapamycin PFC NP treatment reduced VCAM-1 expression 48 h after cisplatin injection. β-Actin served as the loading control. (**F**) Quantification of VCAM-1 expression. ns: not significantly different. (*n* = 3 per group, mean ± SEM).

VCAM-1 staining was performed on the kidneys of mice either 24 or 48 h after cisplatin injections with or without rapamycin PFC NP treatment. Congruent with the ICAM-1 observation, The results illustrated that the VCAM-1 signals from the kidneys of rapamycin PFC NP-treated mice (Figure 5A) was lower than control mice (Figure 5B) at 24 h after cisplatin injection. While the VCAM-1 expression was significantly reduced in treated mice h (Figure 5C) comparing to control mice (Figure 5D) 48 after cisplatin injection. The observation was confirmed by Western blotting (Figure 5E). Twenty-four hours after cisplatin injection, VCAM-1 protein expression normalized to β-actin was 0.03 ± 0.01 for rapamycin PFC NP-treated mice vs. 0.05 ± 0.02 for untreated controls (*p* > 0.05). Forty eight hours after cisplatin injection, VCAM-1 protein expression normalized to β-actin was 0.019 ± 0.003 for rapamycin PFC NP-treated mice vs. 0.083 ± 0.019 for untreated controls (*p* < 0.05), which was a 77% decrease.

### 2.5. Rapamycin PFC NP Treatment Reduced Cell Death

**Figure 6:**
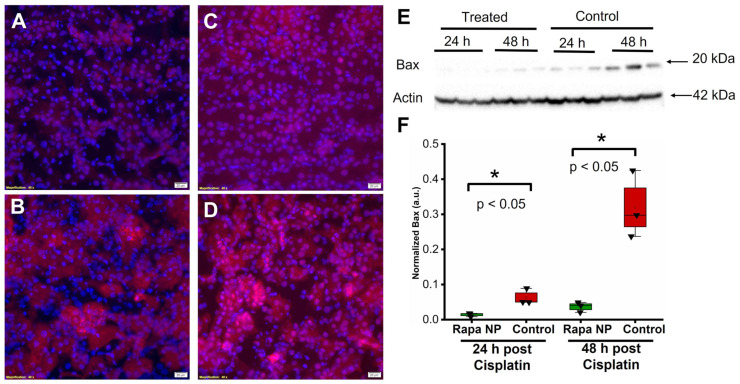
Rapamycin PFC NP treatment reduced Bax expression in the kidney. (**A**,**B**) Representative images of Bax staining (red) in the kidney from mice 24 h after cisplatin injection with (**A**) or without (**B**) rapamycin PFC NP treatment. (**C**,**D**) Representative images of Bax staining (red) in the kidney from mice 48 h after cisplatin injection with (**C**) or without (**D**) rapamycin PFC NP treatment. DAPI nuclear staining is shown in blue. The size of the scale bar is 20 µm and the magnification is 40×. (**E**) Western blot of Bax showing rapamycin PFC NP treatment reduced Bax expression 48 h after cisplatin injection. β-Actin served as the loading control. (**F**) Quantification of Bax expression. (*n* = 3 per group, mean ± SEM).

The induction of apoptosis is one of cisplatin’s off-target effects [28]. Here, we evaluated the effects of rapamycin PFC NP on the expression of the pro-apoptotic protein Bax using immunofluorescent staining and Western blotting. The results illustrated that Bax levels from the kidneys of rapamycin PFC NP-treated mice either 24 h (Figure 6A) or 48 h (Figure 6C) after cisplatin injection were significantly lower than those in untreated control mice 24 h (Figure 6B) or 48 h (Figure 6D) after cisplatin injection. The observation was confirmed by Western blotting (Figure 6E). As demonstrated in Figure 6F, in the kidneys from mice 24 h after cisplatin injection, Bax protein expression normalized to β-actin was 0.014 ± 0.003 for rapamycin PFC NP-treated mice vs. 0.063 ± 0.013 for controls (*p* < 0.05), which was a 78% decrease. At 48 h after cisplatin injection, Bax protein expression normalized to β-actin was 0.014 ± 0.008 for rapamycin PFC NP-treated mice vs. 0.320 ± 0.055 for controls (*p* < 0.05), which was a 96% decrease.

**Figure 7:**
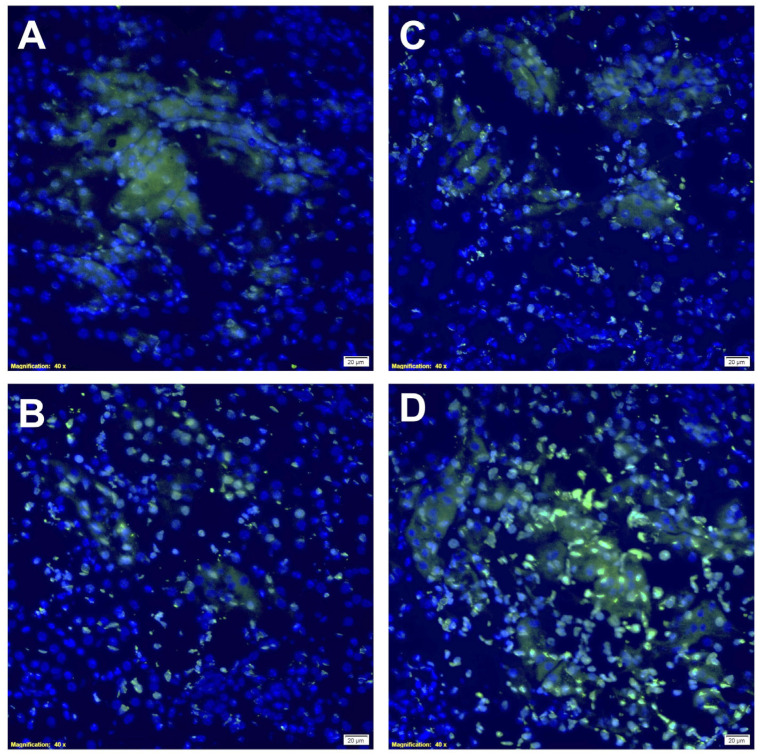
Rapamycin PFC NP treatment reduced DNA damage in the kidney. Representative images of TUNEL staining in the kidney from mice 24 h after cisplatin injection with (**A**) or without (**B**) rapamycin PFC NP treatment. Representative images of TUNEL staining in the kidney from mice 48 h after cisplatin injection with (**C**) or without (**D**) rapamycin PFC NP treatment. Green indicates apoptotic cells. DAPI nuclear staining is shown in blue. The size of the scale bar is 20 µm and the magnification is 40×.

As illustrated in Figure 7, rapamycin PFC NP treatment resulted in reduced TUNEL staining at 24 h (Figure 7A) and 48 h (Figure 7C) after cisplatin injection compared to untreated control mice at 24 h (Figure 7B) and 48 h (Figure 7D) after cisplatin injection, respectively. These results indicated rapamycin PFC NP treatment reduced apoptosis in the kidney injured by cisplatin.

## 3. Discussion

Cisplatin is used to treat various types of cancer, including head and neck cancer [29], ovarian caner [30], and lung cancer [31,32]. The full anticancer potential of cisplatin remains underutilized due to treatment-induced toxicities, including neurotoxicity [33], ototoxicity [34], and nephrotoxicity [35,36,37]. Nephrotoxicity in the form of acute kidney injury is particularly significant in limiting the use of cisplatin because the concentration of cisplatin in proximal tubule cells in the kidneys is five times greater than that in serum [38,39]. The high concentration of cisplatin in proximal tubule cells is due to absorption mediated by Ctr1, a copper transporter, and organic cation transporters (OCTs) expressed on the surface of those cells [40,41]. In this work, we demonstrated that therapy with rapamycin PFC NP can mitigate the deleterious consequences of cisplatin on kidney function in part by enhancing autophagy and suppressing inflammation, particularly in vascular structures.

Compared to free rapamycin, PFC NP serves as a stable depot for the sustained release of rapamycin, thus providing high tissue levels of the drug without needing high serum levels in order to achieve the gradient for rapamycin tissue entry. We have shown this approach to be effective in cardiac pathologies such as muscular dystrophy in the *mdx* mouse model where PFC NP permeated inflamed or damaged tissues and were trapped in interstitial compartments to continuously release high concentrations of rapamycin locally over time [42]. This ingress of PFC NP also occurs in acute kidney injury due to endothelial permeability and retention (EPR) effects subsequent to vascular damage [43,44].

Cisplatin’s adverse impacts on the endothelium play an important role in the underlying pathophysiological mechanism for cisplatin-induced acute kidney injury [45]. Both clinical and pre-clinical studies suggest that cisplatin treatment induces endothelial cell activation, dysfunction, and injury [46,47,48,49]. Cisplatin-induced endothelial activation results in the increased expression of adhesion molecules, such as ICAM-1 [50,51] and VCAM-1 [52], which can contribute to the recruitment and migration of leukocytes into tissues. Additionally, the associated inflammation is fundamental to the initiation and progression of acute kidney injury [53]. Our results indicated that rapamcin PFC NP treatment significantly reduced expression levels of ICAM-1 and VCAM-1 (Figure 4 and Figure 5) 48 h after cisplatin injection, which was well correlated with the kidney function preservation measured by BUN (Figure 2B). The suppression of these key facilitators of immune cell responsivity by rapamycin PFC NP also accorded with the paucity of evidence for tubular injury caused by exposure to cisplatin at 24 and 48 h (Figure 2C,D).

Body weight loss is a common side effect of cisplatin treatment. Patients with head and neck cancer who received cisplatin-based chemotherapy had a significant decrease in body weight and muscle mass [54], but body weight loss was not found to be associated with overall survival [55], which is different from reports on ovarian and lung cancer patients. Ovarian cancer patients receiving cisplatin-based chemotherapy had a significant reduction in body weight and body mass index (BMI), and body weight loss was associated with poor overall survival [56]. Cisplatin-induced weight loss can be a predictor of poor prognosis in lung cancer patients, as those who experienced weight loss during cisplatin treatment had a higher risk of disease progression and death compared to those who did not lose weight [57]. Our results indicated that rapamycin PFC NP treatment significantly reduced cisplatin-induced body weight loss as early as 24 h after cisplatin injection (Figure 2A). Because cancer patients are susceptible to body weight loss from the cancer itself as well as the therapies administered, the reduced body weight loss accompanying rapamycin PFC NP treatment would have benefits in reduced morbidity and mortality. We presume that, in this case, the induction of autophagy could streamline the degradation and recycling of damaged cellular components for maintaining cellular homeostasis and the survival of kidney cells under cisplatin injury.

Autophagy is a process by which cells break down and recycle cellular components. Autophagy also helps cells cope with stress and clear out damaged or aged cellular components to prevent toxic buildup [58]. p62 is a ubiquitin-binding protein that binds to damaged or aged proteins and other cellular components and targets them for degradation. A decrease in the amount of p62 indicates increased autophagic activity. LC3B I and LC3B II are two forms of the protein LC3B, which is involved in autophagy. LC3B I is the inactive form of the protein whereas LC3B II is the active form. LC3B I is cleaved to form LC3B II, which is then involved in the formation and degradation of autophagosomes. An increased LC3B II/I ratio suggests enhanced autophagy. Our results demonstrated that rapamycin PFC NP treatment enhanced autophagy as early as 24 h after cisplatin injection. Consequently, its renal protective effects could be potentially due to the facilitated clearance/degradation of damaged cellular components in the kidney, which can be reprocessed for tissue restoration. Recent preclinical investigations further confirmed the role of autophagy in mitigating cisplatin-induced acute kidney injury. In addition to rapamycin [59], ginsenoside Rb3 [60] and lithium [61] enhanced autophagy through the AMPK signaling pathway and also exhibited renoprotective effects under cisplatin treatment. Moreover, a recent study suggested that rapamycin treatment only achieving mTOR inhibition without autophagy enhancement did not show renoprotection under cisplatin injury [62].

Cisplatin works by binding to and damaging DNA, which triggers a cascade of events leading to cell death [63]. Apoptosis is considered to be a key mechanism of cisplatin-induced cytotoxicity and is important for the drug’s efficacy in treating cancer [64]. Cisplatin-induced apoptosis can also occur in normal cells. Bax is a pro-apoptotic protein that plays a key role in inducing apoptosis in cisplatin-induced acute kidney injury [65]. It has also been reported that Bax is involved in regulating necrotic cell death [66]. TUNEL (TdT-mediated dUTP Nick-End Labeling) staining is a widely used technique for detecting apoptosis by labeling the ends of DNA fragments generated during apoptosis. However, it is important to note that TUNEL staining can also label DNA breaks generated by necrosis and other non-apoptotic mechanisms. Therefore, combining the Bax measurements (Figure 6) and TUNEL staining (Figure 7), our results suggested that rapamycin PFC NP treatment might also protect the kidney from cell death caused by cisplatin-induced necrosis on top of apoptosis.

Perfluorocarbon is a safe, effective, and flexible theranostic tool that has been approved for clinical use as a blood substitute [67,68] as well as in angioplasty [69] and retinal hemorrhage [70]. Our recently published work also demonstrated the favorable safety profile of rapamycin PFC NP [26]. PFC NP is composed of a hydrophobic PFC core and surrounding lipid monolayer [71,72]. The fluorine (^19^F) signature in the PFC core enables PFC nanoparticles to provide a contrast agent for ^19^F magnetic resonance imaging (MRI) and ^19^F magnetic resonance spectroscopy (MRS) in order to detect and quantify delivery of PFC NP to the site of interest [73,74,75]. The hydrophobic lipid monolayer of PFC NP could be functionalized to carry fluorescent molecules, e.g., Rhodamine (Red), to facilitate microscopic localization of PFC nanoparticles. The hydrophobic lipid monolayer of PFC NP could also serve as a carrier for a hydrophobic drug such as rapamycin, which can be stably dispersed and retained in the lipid monolayer of PFC NP. Our previous studies demonstrated that the pharmacokinetics (PK) of rapamycin-loaded PFC NP measured by blood content of rapamycin [26] agreed with that measured by ^19^F MRS [76], which confirmed the stability of rapamycin PFC NP in circulation required to enable sustained local drug delivery. This current demonstration of therapeutic efficacy for mitigating cisplatin-induced acute kidney injury suggests a new formulation and use for an approved agent, rapamycin, to facilitate chemotherapy in patients requiring cisplatin.

## 4. Materials and Methods

### 4.1. Nanoparticle Formulation

The formulation of rapamycin perfluorocarbon nanoparticles was completed using a previously described method [77] with modifications. Briefly, a lipid/rapamycin mixture of 98.6 mol% egg lecithin, 1 mol% dipalmitoyl-phosphatidylethanolamine (Avanti Polar Lipids, Piscataway, NJ, USA), and 0.4 mol% rapamycin (Cat No. J62473, Alfa Aesar via FisherSci, Tampa, FL, USA) was dissolved in a mixture of methanol and chloroform (1:3, *v*/*v*). For the fluorescent perfluorocarbon nanoparticle, the lipid film composition was 98.6 mol% egg lecithin, 0.7 mol% dipalmitoyl-phosphatidylethanolamine, 0.3 mol% 1,2-dipalmitoyl-sn-glycero-3-phosphoethanolamine-*N*-(lissamine rhodamine B sulfonyl) (ammonium salt) (Avanti Polar Lipids, Piscataway, NJ, USA), and 0.4 mol% rapamycin (Cat No. J62473, Alfa Aesar from FisherSci, Tampa, FL, USA). The solvents were removed under reduced pressure to generate a lipid film with rapamycin, which was dried in a vacuum oven overnight. The next day, the lipid film containing rapamycin (2.0%, *w*/*v*), perfluorocarbon (Gateway Specialty Chemicals, St. Peters, MO, USA) (20%, *w*/*v*), and MilliQ water were sonicated and emulsified at 20,000 psi for 6 passes in an ice bath (LV-1 Microfluidics emulsifier; Microfluidics, Newton, MA, USA). For therapeutic applications, perfluorooctyl bromide was used as the hydrophobic core of the perfluorocarbon nanoparticles. For ^19^F magnetic resonance spectroscopy and magnetic resonance imaging, crown ether was the hydrophobic core material for the formulated nanoparticles.

### 4.2. Nanoparticle Distribution and Zeta Potential Measurement

The size distribution of the rapamycin PFC nanoparticles was evaluated by dynamic light scattering (Brookhaven Instruments Corp., Holtsville, NY, USA). The surface charge of the nanoparticles was determined using a PALS Zeta Potential Analyzer (Brookhaven Instruments Corp.). Data were collected in the mode of phase-analysis light-scattering (PALS) after the solution was equilibrated at 25 °C. All samples were diluted in MilliQ water.

### 4.3. Acute Kidney Injury Model

To generate cisplatin-induced acute injury in mice, C57BL/6 mice (Jackson Laboratory, Bar Harbor, ME, USA) at 13 weeks of age were injected intraperitoneally with cisplatin solution at 25 mg/kg. The cisplatin (TSZ CHEM via FisherSci, Tampa, FL, USA) solution of 1 mg/mL was prepared by dissolving cisplatin in sterile saline just before the injection. All animal-related procedures were approved by the Washington University School of Medicine’s IACUC.

### 4.4. Tissue Collection and Preservation

Mice received either rapamycin PFC NP or saline 24 h before cisplatin was administrated intraperitoneally at a dose of 25 mg/kg. Body weight was monitored daily. Either 24 or 48 h post cisplatin administration, mice were euthanized for blood and kidney collection. Whole blood was collected to obtain serum for blood urea nitrogen (BUN) testing. One kidney was preserved by snap-freezing in liquid nitrogen before being stored at −80 °C prior to protein extraction. Another kidney was cut longitudinally into two halves. One half was preserved in 10% formalin (Sigma, St. Louis, MO, USA) for more than 24 h and less than 48 h before it was processed into a paraffin block, and the other half was preserved in O.C.T. (Fisher Scientific, Waltham, MA, USA) and stored at −80 °C before cryosection.

### 4.5. ^19^F magnetic Resonance Spectroscopy and Magnetic Resonance Imaging on 11.7 T

^19^F magnetic resonance spectroscopy of tissues was performed on a Varian 11.7 T scanner utilizing a custom-designed, 0.5 cm, 4-turn solenoid radio-frequency coil and previously published standard procedures [78]. Tissues were placed in Eppendorf centrifuge tubes and scanned together with an internal standard comprising perfluorooctyl bromide in the core of the perfluorocarbon nanoparticles. ^19^F MRS [spin echo sequence; flip angle 90°, TR = 4 s, sweep width = 100 kHz, 2048 points, acquisition time = 0.02048 s, 128 averages] was performed for quantification of PRC NP content in the kidney. ^19^F MRI (TR = 3 s, TE = 2.264 ms, matrix = 32 × 16, FOV = 20 × 10 mm^2^, 1 slice at 8 mm thickness, resolution = 0.625 × 0.625 × 8 mm^3^, 64 averages) was performed to visualize the location of PFC NP in the kidney. ^1^H T2-weighted MRI was performed to register the kidney.

### 4.6. Serum Preparation

Whole blood was collected in Becton Dickinson (BD) Microtainer SST Amber tubes (FisherSci, Tampa, FL, USA) and left undisturbed at room temperature for 30 min to clot. The clot was removed by centrifuging at 2000× *g* for 10 min in a refrigerated centrifuge (Eppendorf, Enfield, CT, USA). After centrifugation, the supernatant (serum) was immediately transferred into a sterile 1.5 mL polypropylene tube (Fisher Scientific, Waltham, MA, USA) by pipetting. During handling, samples were maintained on ice. Serum samples were stored at −80 °C before blood urea nitrogen (BUN) testing.

### 4.7. Blood Urea Nitrogen (BUN) Test

BUN measurements were performed using a Urea Nitrogen (BUN) Colorimetric Detection Kit (Cat No. K024-H5, Arbor Assays, Ann Arbor, MI, USA) following the manufacturer’s directions. Serum samples were diluted 1:100 for the test.

### 4.8. Histology

10% formalin-fixed kidneys were further processed, sectioned, and H&E stained at the Anatomic and Molecular Pathology Core Histology Lab of the Department of Pathology and Immunology, Washington University School of Medicine. Microscopic images of the H&E slides were acquired with an Olympus bright-field microscope outfitted with a DP27 digital camera to record images of the tissue sections at 20× magnification.

Immunofluorescence staining was performed on O.C.T-embedded frozen kidneys, which were sectioned at 8 µm before staining. Kidney sections were fixed in 4% PFA (Thermo Scientific, Waltham, MA, USA) before staining. Primary rabbit antibodies to p62, ICAM-1, VCAMM-1, and Bax (Abcam, Waltham, MA, USA) were applied onto the frozen section for 1 h at room temperature, followed by the secondary anti-rabbit antibody labeled with Alex 594 (Abcam, Waltham, MA, USA), and the tissue section was incubated at room temperature for 30 min. Slides were then mounted using VECTASHIELD antifade mounting medium with DAPI (Vector Laboratories, Newark, CA, USA), before imaging with an Olympus dark-field microscope outfitted with a HAMAMATSU digital camera C11440 at 40× magnification. Double-blind data acquisition was performed on all images. Blinded data analysis was performed on all kidney images by an experienced renal pathologist (J.G.).

### 4.9. Protein Extraction

Complete lysis buffer was prepared by dissolving 200× stock solution of phenylmethylsulfonyl fluoride (PMSF) (Cell Signaling Technology, Danvers, MA, USA) and one tablet of PhosSTOP *EASY*pack (Roche, Basel, Switzerland) in 10 mL RIPA buffer (Thermo Scientific, Waltham, MA, USA). Kidneys were transferred into the Pink Eppendorf Lysis Kit (Next Advance, Troy, NY, USA) and 200 µL complete lysis buffer was added before being placed in a blue storm bullet blender homogenizer using Speed 10 and Time 3 in a cold room, followed by centrifugation at 16,000× *g* for 20 min at 4 °C in a refrigerated centrifuge (Eppendorf, Enfield, CT, USA). Supernatants were collected, aliquoted, and stored at −20 °C before Western blotting. The protein concentration was quantified using the BCA protein assay (Thermo Fisher Scientific, Waltham, MA, USA).

### 4.10. Western Blot

Under reducing conditions, equivalent amounts (50 µg) of total protein were fractionated using sodium dodecyl sulfate–polyacrylamide gel electrophoresis. Membranes were probed with anti-p62 (Cat No. ab91526), anti-LC3BI/II (Cat No. ab221794), anti-ICAM-1 (Cat No. ab222736), anti-VCAM-1 (Cat No. ab134047), anti-Bax (Cat No. ab32503), and anti-beta actin antibodies (Cat No. ab8227) (1:1000 dilution, Abeam, Cambridge, MA, USA). The host of all primary antibodies was rabbit. Membranes were washed and incubated with secondary antibody anti-rabbit HRP (Cat No. ab16284) (1:10,000 dilution, Santa Cruz Biotechnology, Dallas, TX, USA). Bands were visualized using Pierce ECL Western blotting substrate (Thermo Fisher Scientific, Waltham, MA, USA) with a ChemiDoc MP (Bio-Rad Laboratories, Hercules, CA, USA). Knockdown of proteins was quantified using ImageJ (National Institutes of Health, Bethesda, MD, USA).

### 4.11. TUNEL Staining

TUNEL Staining was performed with the In Situ Cell Death Detection Kit, Fluorescein (Roche, Basel, Switzerland) following user instructions.

### 4.12. Statistics

Results were expressed as the mean ± standard error of mean (SEM). ANOVA with the Scheffé test, *t*-test, and nonparametric analysis were used for statistics. Statistical significance of differences was attributed at *p* < 0.05.

## 5. Conclusions

In conclusion, this study has shown that rapamycin PFC NP can mitigate cisplatin-induced damage in a preclinical mouse model, suggesting that it may serve as a viable prophylactic therapy for patients undergoing cisplatin treatment. However, in order to advance the development of this new therapy for clinical use, further research is needed to investigate the effects of rapamycin PFC NP treatment at different stages of cisplatin injury in preclinical settings. This information could be helpful to identify potential measures for patient stratification and define inclusion and exclusion criteria for designing clinical trials.

## Data Availability

The data presented in this study are available in within the article.
